# Exploring the Impact of Obesity on Health Care Resources and Coding in the Acute Hospital Setting: A Feasibility Study

**DOI:** 10.3390/healthcare8040459

**Published:** 2020-11-04

**Authors:** Winnie S. Y. Tan, Adrienne M. Young, Alexandra L. Di Bella, Tracy Comans, Merrilyn Banks

**Affiliations:** 1 School of Exercise and Nutrition Sciences, Faculty of Health, Queensland University of Technology, Brisbane 4059, Australia; 2 Department of Nutrition and Dietetics, Royal Brisbane and Women’s Hospital, Brisbane 4029, Australia; Adrienne.Young@health.qld.gov.au (A.M.Y.); alexandra.dibella@health.qld.gov.au (A.L.D.B.); Merrilyn.Banks@health.qld.gov.au (M.B.); 3 Centre for Health Services Research, The University of Queensland, Brisbane 4067, Australia; t.comans@uq.edu.au

**Keywords:** obesity, body mass index, hospitals, hospital costs, health care costs, inpatients

## Abstract

Obesity is costly, yet there have been few attempts to estimate the actual costs of providing hospital care to the obese inpatient. This study aimed to test the feasibility of measuring obesity-related health care costs and accuracy of coding data for acute inpatients. A prospective observational study was conducted over three weeks in June 2018 in a single orthopaedic ward of a metropolitan tertiary hospital in Queensland, Australia. Demographic data, anthropometric measurements, clinical characteristics, cost of hospital encounter and coding data were collected. Complete demographic, anthropometric and clinical data were collected for all 18 participants. Hospital costing reports and coding data were not available within the study timeframe. Participant recruitment and data collection were resource-intensive, with mobility assistance required to obtain anthropometric measurements in more than half of the participants. Greater staff time and costs were seen in participants with obesity compared to those without obesity (obesity: body mass index ≥ 30), though large standard deviations indicate wide variance. Data collected suggest that obesity-related cost and resource use amongst acute inpatients require further exploration. This study provides recommendations for protocol refinement to improve the accuracy of data collected for future studies measuring the actual cost of providing hospital care to obese inpatients.

## 1. Introduction

Over half of the current population is either overweight or obese, with current obesity rates nearly tripling since 1975 [1]. The prevalence of obesity is also significant in Australia, where about one-third of the current adult population is obese [2]. This is a major public health concern as obesity is a known risk factor for various comorbidities including diabetes [3,4], non-alcoholic fatty liver disease [5,6], and cardiovascular diseases [7]. As a result, there is an increasing economic burden on the health care system [8,9]. In fact, approximately 11% of hospital admissions in Australia are estimated to be related to obesity [10].

There is limited research to date on the costs attributable to obesity in the acute hospital setting. Available evidence determined that health care costs and length of stay (LOS) of patients are positively associated with increasing body mass index (BMI) [10,11,12,13,14,15]. However, amongst these studies there are significant differences in the methodologies used and aspects of health care cost investigated. For example, Bahia et al. [11] and Lehnert et al. [15] calculated total inpatient costs attributable to obesity using the “top-down” approach (i.e., taking overall expenditures for each element at the central level to estimate unit costs using formulae [16,17]), while Lal et al. [14] used the ”bottom-up” approach (i.e., using detailed expenditure data at the service provider level to evaluate unit costs [18,19]). From an acute hospital setting perspective, the “bottom-up” approach likely provides a more accurate representation of inpatient costs as it captures changes in treatment costs and LOS attributed to obesity-related comorbidities [15,19]. In terms of aspects of health care costs investigated, Korda et al. only investigated hospitalisation costs [10], whereas Buchmueller et al. also investigated pharmaceutical, emergency room visit and outpatient costs [12]. Due to the different components of direct costs investigated, it is difficult to make comparisons between studies to establish the cost of obesity in the acute care setting [8]. It could be expected that obesity may also increase costs related to equipment (e.g., need for hoists, bariatric beds and wheelchairs) and staffing (e.g., additional time or staff members to support mobilisation or other activities of daily living) [20]. However, to date, there are no studies known to have considered equipment costs or staffing time attributable to obesity when estimating health care costs. This lack of consistency and availability of evidence makes it challenging to quantify the specific aspects of hospital care that may cost more for patients with obesity.

In Australia, obesity is coded in accordance with the Australian Refined Diagnosis Related Group (AR-DRG) [21]. The AR-DRG is a classification system whereby specific DRG codes are used to group inpatients based on clinical conditions requiring similar amounts of hospital resources for treatment and care provision. In Australia, accurate clinical coding is essential for public hospitals to receive funding allocations from the Commonwealth Government [22]. A recent cross-sectional audit in three tertiary hospitals in Brisbane, Australia found that obesity coding was correct in only half of inpatients classified as obese by BMI [23]. Poor coding of obesity has also been identified in other countries, principally the United States of America [24,25,26]. With the growing prevalence of obesity in Australia and the associated health care costs, it is important to investigate the sufficiency of hospital funding received through current obesity coding practices.

A study protocol was designed to explore the impact of obesity on health care costs and accuracy of coding in the acute hospital setting in a sample of inpatients with and without obesity. Specifically, the objectives of this protocol are to: (1) test the hypothesis that the total cost of acute hospital admission is higher for patients with obesity, (2) test the hypothesis that the total cost of hospital admission with activity-based funding revenue dollars for admission is higher for patients with obesity, (3) identify “profit” or “loss” of hospital resource allocations for patients with and without obesity, and (4) determine the accuracy of the current obesity coding practice for patients with obesity. This paper provides a detailed outline of a study protocol including data collection timing and procedures for all data (i.e., demographic data, anthropometric measurements, clinical characteristics, health care cost and coding data).

The primary aim of this study is to test the feasibility of this protocol in terms of process, resources required and variance in cost outcomes, with the purpose of informing protocol refinement and sample size calculations before undertaking a larger study. This paper provides a detailed overview of the proposed protocol and reports the feasibility of undertaking this study in a small sample within a single hospital ward.

## 2. Materials and Methods

This prospective observational cohort study was conducted over three weeks in June 2018 in a single orthopaedic ward of a 1000-bed metropolitan tertiary hospital in Queensland, Australia. This ward was chosen for the feasibility study due to higher than average prevalence of obesity in a recent hospital audit.

### 2.1. Participants

Participants were adults aged 18 years and above and had a length of stay (LOS) of less than four days at the time of recruitment. This was chosen to maximise participant retention rate because the average LOS in Australian public hospitals is 5.7 days [27]. Participants were excluded if they were pregnant, had an eating disorder, had cognitive and/or intellectual impairment(s) precluding their ability to provide informed consent or understand data collection procedures, or were discharged prior to the completion of data collection on the observation day. Participants were consecutively recruited over 12 weekdays (within a three-week period) in June 2018. For this feasibility study, there was no pre-determined sample size target. Participants were provided written information (concise two-page summary of study requirements) and verbal explanation from the research assistant (W.S.Y.T., final year nutrition and dietetics student). They were informed that participation was voluntary, made aware of the possibility to withdraw from the study at any time, and understood that hospital care provision would not be affected regardless of participation in this study. The research assistant returned at a time negotiated with the patient to answer questions and obtain written informed consent. This study was approved by the hospital Human Research Ethics Committee (HREC/18/QRBW/67).

### 2.2. Data Collection

Data were collected on patient demographics (age, sex, comorbidities using the Charlson Comorbidity Index (CCI) [28]), anthropometry (weight, height, waist circumference (WC)), and functional status (Katz index of independence with Activities of Daily Living (Katz ADL) [29]), as summarised in Table 1. Where patients were not able to mobilise independently, physiotherapy and/or nursing staff assisted with mobilising patients to allow anthropometric measurements to be taken.

Cost of staffing and equipment were estimated through direct observation of patient care and equipment needs on a single day of admission, as summarised in Table 1. A daily maximum of five participants were chosen for observation each day. On days where there were more than five eligible participants recruited, those with longer LOS or expected to be discharged soon were prioritised to maximise participant retention rate. The observation procedure was based on behavioral mapping procedures by Kuys et al. [30], whereby each participant was observed for 1 min in every 10-min interval from 7.30 a.m. to 5.30 p.m. This means 55 separate observations were undertaken for each participant over a 10-h period. At each 10-min observation interval, the presence of staff was recorded as 10 min of staff–patient interaction. Staffing time was estimated to the nearest 10 min. The duration of staff–patient interaction is cumulative, i.e., when the same staff was present at two subsequent observations periods, the staff–patient interaction duration was documented as 20 min. Equipment use was noted at each 10-min interval, with cost obtained from the hospital procurement services. Photographs of equipment were taken to ensure correct classification and costing. All data were collected by a single research assistant (W.S.Y.T.).

Cost of hospital encounter and activity-based funding revenue dollars for each participant were obtained from costing reports provided by the hospital finance department. These reports included the following data: (1) breakdown of cost of hospital encounter (bed-day cost, allied health, imaging, pathology, patient support services, pharmacy, surgery doctor, surgery theatre, outpatient clinic), (2) primary diagnosis, and (3) allocated activity-based funding revenue dollars. It is important to note that, while staffing costs are included in these reports, these are based on estimates (i.e., not based on delivered care) and are not adjusted based on impairment or obesity. Obesity coding data were obtained from the health information management department. As per usual practice, administrative officers in the health information management department allocate patients with a code for obesity where evidence that the Australian Coding Standard (ACS) 0002 for Additional Diagnoses has been met; that is, there is evidence in the medical chart demonstrating that obesity affected patient management in terms of requiring any of the following: commencement, alteration or adjustment of therapeutic treatment; diagnosis procedures; or increased clinical care and/or monitoring [31]. A BMI of 30 or above is not sufficient to code a patient as obese without evidence of change in care.

The feasibility of the data collection procedures was assessed in terms of “process” and “resources” as outlined in Table 2. ”Process” refers to the feasibility of fundamental protocol procedures such as participant recruitment and data collection, while “resources” refers to the feasibility of time, human resources and equipment required for the study [32].

### 2.3. Statistical Methods

Descriptive statistics were used to report feasibility, staff time, equipment costs and patient data (characteristics, anthropometry and functional status). Histograms were used to assess normality of distribution, with medians and interquartile range reported where data were assessed as non-normal. Staffing cost was estimated using the equation: number of staff present × allocated cost of staff per hour (AUD) × duration of interaction with patients (hours). The allocated cost of staff per hour was based on current Queensland Health wage rates (accessed on 29 June 2018) [39]. BMI was calculated for each participant using weight (kg) divided by height (m) squared. The World Health Organization BMI classifications were used: underweight (<18.5 kg/m^2^), normal weight (18.5–24.9 kg/m^2^), overweight (25–29.9 kg/m^2^), obese I (30–34.9 kg/m^2^), obese II (35–39.9 kg/m^2^), obese III (≥40 kg/m^2^) [40]. WC was classified as overweight at >94 cm (men) and >80 cm (women), and obese at >102 cm (men) and >88 cm (women) [36].

Staffing and equipment costs were compared between participants with and without obesity (based on BMI) using independent-samples *t*-test and chi-square test of independence. Analyses were conducted using IBM SPSS Statistics for Windows, Version 25.0 (IBM Corp., Armonk, NY, USA). Due to the small sample size and study aims related to feasibility (rather than hypothesis testing [41]), tests for statistical significance have not been conducted; instead, these data are presented to explore averages and variance to inform the design of future studies.

## 3. Results

**Figure 1:**
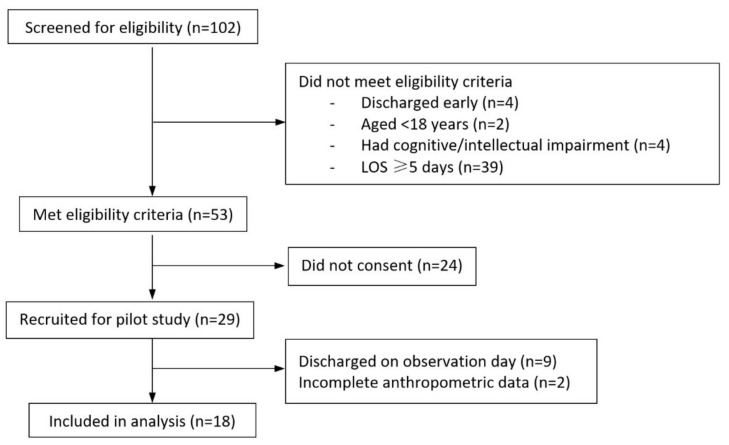
Participant flow during the 3-week feasibility study. LOS, length of stay.

Over three weeks (12 weekdays), 102 inpatients were screened and 29 were recruited (recruitment rate of 28%, consent rate of 55%, daily average of 3 (SD: 1.6) participants). Twenty participants remained as inpatients throughout observation days, but only 18 complete sets of participant demographic and clinical data were obtained (retention rate of 62%). The participant flow during the feasibility study is shown in Figure 1. Participant LOS of more than 4 days at time of recruitment was the most common reason for participant ineligibility. Early discharge before completion of recruitment and data collection was the most common reason for reduced retention rate. Table 3 summarises the amount of missing data for each data item. Gold standard measurements were completed for waist circumference in 15 participants (83%), weight in 14 participants (78%) and height in eight participants (44%). Mean BMI was 28.7 kg/m^2^ (SD: 7.7, range: 19.1–45.9 kg/m^2^); with six participants (33%) classified as obese according to BMI and nine (53%) classified as obese according to WC.

The total time taken to recruit participants and collect “Pre-Observation” data was approximately 40 h, equivalent to 1 h and 20 min per participant. From this, the estimated time taken to recruit each participant was 20 min. This includes the time spent providing participants with both written information and verbal explanation of the study, addressing any questions, and obtaining the written informed consent. The remaining one hour spent collecting “Pre-Observation” data from each participant was inclusive of the time required to obtain assistance from physiotherapists and/or nursing staff to mobilise participants. A further ten hours of time was spent collecting observational data on each observation day. Ten (56%) participants required mobility assistance, and none of their anthropometric data could be collected on the day of recruitment. This was largely attributed to participants preferring to mobilise only during their physiotherapy sessions, which were fixed according to the physiotherapists’ daily schedules. All equipment for anthropometric data collection were available and accessible when required.

The estimated mean time that staff were observed to spend directly with patients during the 10-h study period was 17.5 h (SD: 7.8, range: 7.0–32.4), with nurses and physiotherapists spending the most time with patients (total 157 h and 67 h, respectively). Using these data, mean staffing cost per day per participant was calculated to be AUD 131.04 (SD: 64.24, range: 45.99–259.40). Majority of the total staffing cost was attributable to nursing (AUD 971.64), physiotherapy (AUD 593.73) and ward round team (generally consists of physicians, nurses and medical students) (AUD 298.65). Data were collected on the type of equipment used for all participants. However, estimating equipment cost was not possible due to the number of different types of mobility aids used by individual participants on the observation day, making it difficult to determine an average cost. Furthermore, no bariatric-specific equipment was used by recruited participants. Observations and clarification with physiotherapists and nurses found that the choice in type of equipment was generally determined by functional impairment or clinical conditions (e.g., traction frame was used for a participant with hip fracture) rather than obesity-related reasons. Data on primary diagnosis, cost of hospital encounter, activity-based funding revenue dollars and assigned obesity DRG codes were not available at the time of data analysis (four months after study period). Table 2 summarises the feasibility data in terms of process and resources required.

Data comparing staffing time and costs, and characteristics of patients with and without obesity are displayed in Table 4. From this pilot data, participants with obesity had reduced functional status and increased staffing cost compared to those without obesity. Conversely, participants without obesity appeared to have a higher CCI compared to those with obesity.

## 4. Discussion

This study was conducted to determine the feasibility of recruiting and collecting data from acute hospital inpatients, in order to investigate obesity-related health care costs and coding. The average daily participant recruitment rate of three participants was less than the daily target of five participants. To ensure sufficient sample size for meaningful data analysis, the data collection period may need to be extended and/or conducted in more than one ward. Further consumer consultation may be required to identify additional strategies to improve consent rates in future studies; current evidence is limited to clinical trials [42] and is not applicable to pragmatic observational studies such as this. While the participant retention rate is relatively low due to early discharge from the hospital, it is recommended that the current eligibility criteria regarding LOS is maintained and not reduced from “less than four days at time of recruitment”. This is because the main reason for participant ineligibility was their LOS exceeding this eligibility criteria, i.e., having a LOS of five days and greater.

Most of the required “Pre-Observation” data (Table 1) were obtained. Missing data were due to resource issues; particularly, the short data collection period and challenges in obtaining timely assistance from physiotherapists and/or nursing staff to mobilise participants resulted in delayed or missing data. For example, participants required support to be positioned upright for WC measurements due to limited mobility. A consensus statement from obesity experts suggested that WC is redundant if BMI was already calculated [43]. However, several studies have determined WC as an independent indicator of obesity-related health care costs and morbidity, whereby the addition of BMI did not improve or alter study findings [40,44,45]. Thus, it is unclear if BMI and WC should be used together or separately to investigate obesity-related cost and health implications. For future studies, it is recommended that both measurements continue to be obtained; but only in wards where recruited participants are unlikely to have significant mobility limitations that will affect the accuracy of WC measurements. Data from costing reports and coding data (Table 1) were inaccessible at time of data analysis, which was four months after conducting the feasibility study. Despite estimated availability approximately six to eight weeks post-discharge, these data were significantly delayed. To avoid this in future studies, additional time (e.g., up to six months) may need to be factored into study timelines to ensure availability of all data. Although the cost of equipment was obtained from hospital procurement services, this was not included in the analysis due to multiple types of equipment used by individual participants through the course of a single day. Future data collection may be better focused on bariatric versus non-bariatric equipment use, due to the known significant cost difference. Given the low use of bariatric equipment and choice of equipment based on functional impairment or clinical conditions, this study suggests that it may be possible that equipment does not significantly add to health care costs of obese inpatients; however this requires testing in future studies. It may also be important to investigate any differences in ambulatory difficulties, and therefore mobility equipment utilisation, between those with and without obesity.

The research assistant and equipment resources allocated were sufficient. However, there were difficulties in obtaining timely assistance from physiotherapists and/or nursing staff to mobilise participants. As more than half of the recruited participants required mobility assistance, it is recommended that physiotherapists and/or nursing staff of selected wards are included as part of the project in future studies. Extra time allocation and/or training for the research assistant to complete this task independently may also be strategies to improve collection of this data in the future. The use of the activity mapping methodology by Kuys et al. [30] was not without limitations. Occasions where participants required assistance behind privacy curtains or closed doors (e.g., changing clothes or toileting using bedpans) were not documented. These were potentially valuable missing data as the amount and/or duration of staff assistance required may have significantly altered total staff time and therefore cost. Some participants were also off-ward for up to half a day for procedures or appointments. Accumulatively, these have resulted in limited observation data, and likely explains the low estimated average staffing cost. These issues were not discussed by Kuys et al. [30], the study from which this observation protocol was based on. Considering the unpredictable differences in: (1) individual characteristics of both inpatients and health professionals, (2) inpatients’ clinical needs, and (3) necessary privacy in specific occasions, observation alone may not provide comprehensive data to estimate staffing cost, despite the resource-intensive process. Hence, it is recommended that further investigations are conducted to explore alternative methods currently used or tested effective in determining hospital workforce requirements. Amongst health professionals observed in this study, nursing staff and physiotherapist requirements were seen to contribute most to patient care. Therefore, it may also be useful to conduct qualitative surveys with the nursing staff and physiotherapists to investigate if there are substantial differences in time and number of staff requirements specifically due to obesity. Another consideration is the use of real-time location systems (RTLS) to track staff real-time locations and time spent at the patient bedside. This may improve the accuracy of estimated staff–patient interaction, particularly during private occasions where observation data cannot be collected. However, this will also require additional considerations to be made, including the choice of RTLS technology suitable for the study site, cost of implementation, interference concerns, patient and staff acceptability, and technological limitations [46,47,48].

Results from the bivariate analysis should be interpreted with caution considering the small sample size (*n* = 18). Staffing time and cost appeared greater for participants with obesity, however the significant variability between groups (as indicated by the large SD) indicates the unreliability of findings from this pilot data. To determine accurate cost estimates, this needs to be re-investigated using refined methods and with a larger sample size. Based on the cost data collected from this feasibility study, it is estimated that a sample size of 52 participants (26 per group) is needed to detect a significant difference in cost outcomes between obese and non-obese inpatients with 80% power, using a two-sample t-test and assuming an α of 0.05.

Despite the limitations with the accuracy and completeness of data collection, this study is the first to explore and describe challenges with costing obesity at the ward level. Moreover, this study highlights a number of considerations for researchers attempting to quantify the cost of obesity for acute inpatients. Overall, important modifications to the study protocol were determined, providing a clear direction for future studies.

## 5. Conclusions

It is challenging to measure and quantify the cost and impact of obesity on staff time in the acute hospital setting. Inpatients with obesity appear to incur increased costs related to greater staff time when compared to those without obesity. However, these findings are likely to be underrepresented in current methods. Despite these limitations, it is imperative to continue to explore similar investigations as current evidence emphasises the significant economic burden of obesity on health care systems. By identifying specific aspects of inpatient cost that increase due to obesity, important targeted cost-saving strategies can be implemented by hospitals to address this economic burden effectively and ensure appropriate remuneration. Obesity coding practice, which has not been successfully investigated in this study, should also be prioritised in future studies as it informs the fundamental hospital funding allocation attributable to obesity. Additional evidence on obesity-related hospitalisation costs may highlight the need for reinvestigation of obesity clinical coding standards and practices. These increased insights into the economic burden of obesity can benefit health economists and policy makers, as they will be able to advocate for policy refinements and resource reallocations for the adequate provision of care to inpatients with obesity. This feasibility study forms the foundation by informing essential modifications of the study protocol to improve the accuracy and success of investigating obesity-related hospital costs in future studies. Particularly, considerations of innovative methods to ensure more robust capture of actual staffing requirements.

## Figures and Tables

**Table 1:** Summary of data collection procedures for each consented participant.

Day/Timeline	Variables	Data Collection Procedures
Pre-Observation	Age Sex CCI score Height Weight BMI WC Katz ADL score	Review medical records (medical charts and/or ward charts) for: Age Sex Comorbidities to calculate CCI score Complete Katz ADL questionnaire with participant Collect anthropometric data. Height and weight measurement methods are listed in order of priority. Chosen measurement methods were dependent on the participant’s mobility as advised by senior ward physiotherapist: Height Using stadiometer (gold standard) Estimated using knee height (using procedures and equations outlined by L’her et al. [33]) Estimated using ulna length (using procedures and equations outlined by Barbosa et al. [34]) Self-reported or obtained from medical records Weight * Using digital or chair scale (gold standard) Self-reported or obtained from medical records Estimated using measured height and arm circumference (using procedures and equations outlined by Crandall et al. [35]) BMI: weight (kg) divided by height (m) squared WC: Measured with patient upright and upon exhalation, at midpoint between bottom of last palpable rib and top of hip bone or at the level of navel [36,37]
Observation day (7.30 a.m.–5.30 p.m.)	Duration of staff–patient interaction Equipment used	Observe each participant for 1 min at every 10-min interval (based on procedures outlined by Kuys et al. [30]) - Observe from a distance, i.e., outside the doorway or along the corridor - Do not observe if privacy curtains are pulled, door closed, or when participant is off-ward - As required, clarify discipline of staff interacting with participant and types of equipment used by participant; take photos of equipment used to allow accurate classification by procurement services Observation breaks (50 min total): 2 × 10 min (9.30 a.m., 3.00 p.m.), 1 × 30 min (12.30 p.m.–lunch break)
Post-discharge	Primary diagnosis Cost of hospital encounter Activity-based funding revenue dollars Assigned DRG code for obesity Cost of equipment	Request for participant’s costing report from district finance department Request for coding data from health information management department Request for equipment cost data from procurement services

* For participants with amputations, their weights were corrected using Durkin et al.’s calculations [38]. CCI, Charlson Comorbidity Index; BMI, body mass index; WC, waist circumference; Katz ADL, Katz index of independence with Activities of Daily Living; DRG, Diagnosis Related Group.

**Table 2:** Summary of study feasibility objectives, measures and results.

Aspects	Objectives	Feasibility Measures	Feasibility Results
Process	To recruit sufficient participants To obtain all required data (as per Table 1)	• Participant recruitment rate	• 28% (29 of 102 participants)
		• Participant consent rate	• 55% (29 of 53 participants)
		• Participant retention rate	• 62% (18 of 29 participants)
		• Effectiveness/suitability of data collection tool	• All data during “Pre-Observation“ and “Observation“ periods were collected
		• Amount of missing data for each variable	• See Table 3
		• Availability of costing report	• Not available at four-months post-study
		• Availability of obesity coding data	• Not available at four-months post-study
		• Availability of equipment cost data	• Available from hospital procurement services
Resources	To determine the level of research assistant resource required to recruit patients To determine the level of research assistant resource required to collect all required data To determine level of physiotherapy and/or nursing staff resource required to provide mobility assistance To determine the accessibility of equipment required to collect anthropometric data	• Average time to recruit each participant	• 20 min
		• Average time to collect data for each participant	• 11 h (“Pre-Observation“ and “Observation day“ data)
		• All data collected within allowed time	• Costing report and obesity coding data unavailable within study timeframe
		• Percentage of participants requiring mobility assistance; time of required assistance from physiotherapists and/or nursing staff to mobilise participant	• 56% (10 of 18 participants); time required included in participant recruitment time as described above
		• Availability of required equipment	• Available when required

**Table 3:** Summary of available data for participants with completed observations (*n* = 18) at completion of study period.

Variable	*n* (%)
Age	18 (100%)
Sex	18 (100%)
Comorbidities (CCI score)	18 (100%)
Activities of Daily Living	18 (100%)
Weight	18 (100%)
Measured	14 (78%)
Self-reported	4 (22%)
Height	18 (100%)
Stadiometer	8 (44%)
Knee Height	8 (44%)
Ulna	2 (12%)
Waist circumference	18 (100%)
Standing	15 (83%)
Lying	3 (17%)
Primary diagnosis	0
Assigned DRG code for obesity	0
Cost of hospital encounter	0
Cost of equipment	0

CCI, Charlson Comorbidity Index; DRG, Diagnosis Related Group.

**Table 4:** Participant characteristics and staffing cost amongst those with and without obesity.

Variables	Non-Obese (*n* = 12)	Obese (*n* = 6)
BMI categories; count (%)	Normal weight: 7 (39) Overweight: 5 (28)	Obese class I: 2 (11) Obese class II: 2 (11) Obese class III: 2 (11)
Sex, male; count (%)	4 (33)	3 (50)
Age, years; mean (SD)	50.3 (13.9)	52.0 (10.6)
CCI score; median (IQR)	1 (1.3)	0.5 (1)
Katz ADL score; median (IQR)	4 (4)	2 (2)
Staff time (hours); mean (SD)	15.5 (6.7)	21.7 (8.8)
Staff cost; mean (SD)	AUD 113.61 (54.35)	AUD 165.88 (73.13)

BMI, body mass index; CCI, Charlson Comorbidity Index; Katz ADL, Katz index of independence with Activities of Daily Living; SD, standard deviation; IQR, interquartile range.
